# Hepatitis C Presenting Like Endocarditis in a Young Patient With Substance Use Disorder: A Case Report

**DOI:** 10.7759/cureus.64174

**Published:** 2024-07-09

**Authors:** Mohit Chhatpar

**Affiliations:** 1 Family Medicine, Indiana Regional Medical Center, Indiana, USA

**Keywords:** drug-induced hypokalemia, abnormal presentation, substance use disorder (sud), valvular endocarditis, hepatitis c (hcv) infection

## Abstract

This case report details a 21-year-old male patient who initially presented with endocarditis-like symptoms but ultimately had hepatitis C in the setting of substance use disorder. It highlights the value of prompt diagnosis and effective treatment. He had a medical history of chronic heroin use over two years and presented inconsistently to the emergency department with generalized body aches. He had generalized body pain and right upper and lower quadrant abdominal pain. He had been unable to tolerate any oral intake and had been vomiting after every meal for the last three weeks. Physical examination was significant only for large, ovoid, erythematous nodules on the left dorsal foot, blanching and slightly painful to touch; diffuse scabs and sores on extremities; and nodules on dorsal interphalangeal joints on the left hand. Urine drug screen was noted to be positive for cannabinoids, methamphetamines, and opioids. The initial electrocardiogram did not show typical T wave flattening changes for hypokalemia. Transthoracic echocardiogram ruled out infective endocarditis, with no valvular vegetation. He was ultimately found to be hepatitis C virus antibody positive. This case illustrates the importance of keeping a wide differential in mind. The patient had hepatitis C despite being asymptomatic throughout presentation-keeping. The patient’s history of heroin use was critical while ordering testing.

## Introduction

Substance abuse is a growing problem in the United States and worldwide. Commonly abused substances include alcohol, cannabis, opioids, and nicotine. Opioid use is particularly prevalent. According to the World Health Organization, in 2021, approximately 60 million people used opioids, resulting in 80,000 fatalities [1]. During the COVID-19 pandemic, there was an increase in drug overdose deaths reported in the United States, largely due to synthetic opioids [2]. We hereby describe a patient with daily heroin use who presented to the emergency department (ED) with nausea and vomiting. He was admitted for treatment of severe metabolic alkalosis with hyponatremia and hypokalemia but was ultimately also found to have hepatitis C.

## Case presentation

A 21-year-old male presented to the ED, reporting daily heroin use over the past three weeks. He has a history of using heroin for one to two years. He stopped for one year and is now on Suboxone maintenance treatment. However, he stopped taking Suboxone two months ago and started using heroin again, both through IV and insufflation use. For the last two weeks, before presenting to the ED, he had been using heroin once daily.

On presentation to the ED, the patient complained of generalized body pain, as well as pain in the right upper and right lower quadrants of the abdomen. He mentioned that he had used heroin the previous night. He noted that for the last three weeks, since the onset of daily heroin use, he had been unable to tolerate any oral intake and had been vomiting after every meal. He denied chest pain, headache, fever, chills, dizziness, lightheadedness, shortness of breath, hematemesis, coffee-ground emesis, diarrhea, black or bloody bowel movements, recent trauma, recent falls, recent illnesses, or sick contacts.

**Figure 1:**
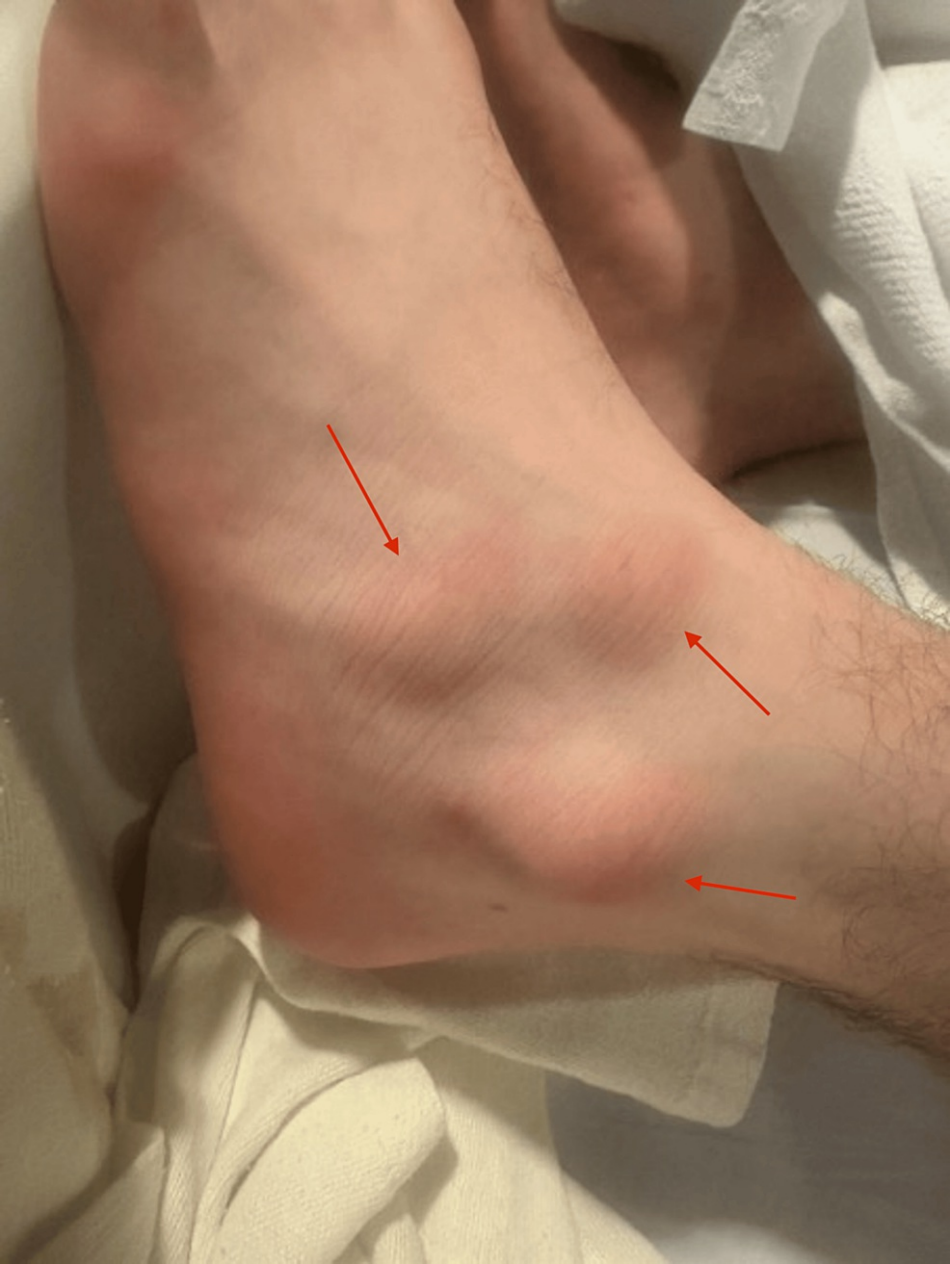
Erythematous nodules in the left foot (red arrows)

**Figure 2:**
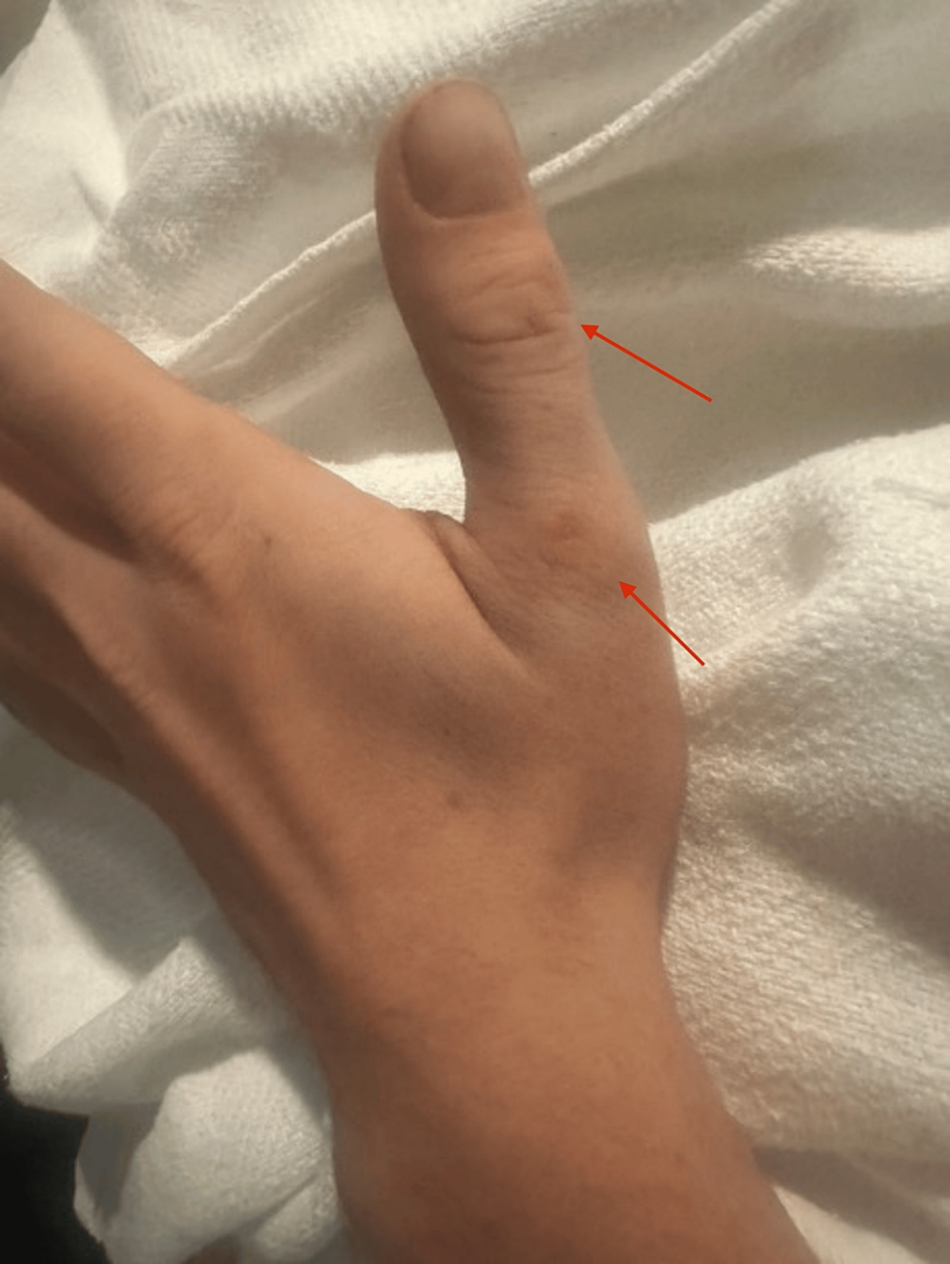
Nodules in the left hand (red arrows)

**Figure 3:**
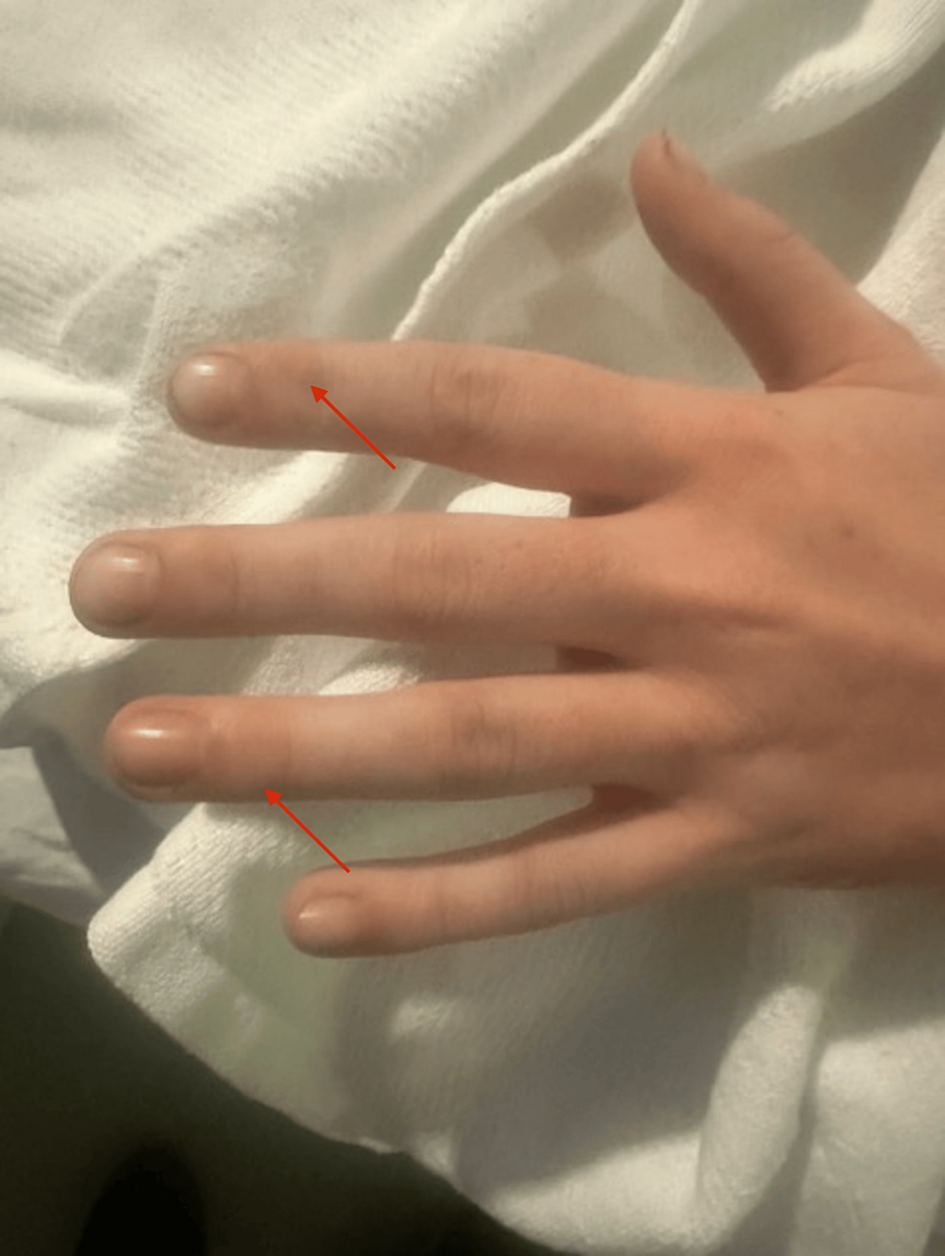
Nodules on DIP joints in the left hand DIP: distal interphalangeal joint

In the ED, vitals were as follows: temperature: 36.8 °C; heart rate: 82/min; respiratory rate: 22 breaths/min; blood pressure: 147/94 mmHg; SpO_2_: 100%; weight: 77.1 kg (measured). Physical examination was significant only for large, ovoid, erythematous nodules on the left dorsal foot (Figure 1), blanching and slightly painful to touch; diffuse scabs and sores on extremities; and nodules on dorsal interphalangeal joints on the left hand (Figures 2, 3). Neurological examination showed that he was alert and oriented × 3, with clear and articulate speech and no gross motor deficits or focal weakness.

Table 1 shows the significant laboratory values on admission.

**Table 1:** Admission lab work

Blood work	Value
White blood cells	14.6 × 10^9^ cells/L (reference: 4.0 × 10^9 ^to 10.0 × 10^9^ cells/L)
Platelets	532,000 cells/mcL (reference: 150,000-350,000 cells/mcL)
Sodium	118 mEq/L (reference: 136-145 mEq/L)
Potassium	2.2 mEq/L (reference: 3.5-5.0 mEq/L)
CO_2_	47 mEq/L (reference: 23-26 mEq/L)
Glucose	129 mg/dL (reference: 70-99 mg/dL)
Creatinine	1.21 mg/dL (reference: 0.8-1.3 mg/dL)
Calcium	9.7 mg/dL (reference: 9.0-10.5 mg/dL)

Urine drug screen results showed positive results for cannabinoids, methamphetamines, and opioids. The patient tested negative for influenza A, influenza B, and COVID-19. The initial electrocardiogram (EKG) did not show typical T wave flattening changes for hypokalemia. He received 1 L of normal saline bolus, 4 mg of ondansetron IV push, and 10 mEq of potassium chloride to replete potassium. The patient's chest X-ray showed increased bronchial markings but no acute infiltrates.

The patient was admitted to the ICU for the treatment of severe metabolic alkalosis due to protracted vomiting, hypochloremia, hypokalemia, and likely hypovolemic hyponatremia. While in the ICU, he was placed on continuous cardiac monitoring and pulse oximetry. On day 1 of admission, a transthoracic echocardiogram ruled out infective endocarditis as there was no valvular vegetation; the patient had a preserved ejection fraction of 55%-60%. An infectious disease expert's opinion was sought, and blood cultures were obtained (Table 2).

**Table 2:** Day 1 laboratory values

Blood work	Value
Sodium	123 mEq/L (reference: 136-145 mEq/L)
White blood cells	9.5 × 10^9^ cells/L (reference: 4.0 × 10^9^ to 10.0 × 10^9^ cells/L)
Potassium	2.2 mEq/L (reference: 3.5-5.0 mEq/L)
Calcium	8.3 mEq/L (reference: 9.0-10.5 mg/dL)

A repeat EKG showed U waves, as expected for hypokalemia. Blood pressure normalized to 126/81 mmHg, and the patient continued to saturate well at 97% oxygen on room air. The patient was again given 10 mEq potassium chloride for repletion, with a target potassium of greater than 4.5 mEq/L (reference: 3.5-5.0 mEq/L). Given urine was positive for cannabis use, it was questioned if cannabis hyperemesis syndrome may be playing a role. The patient was started on buprenorphine and placed on Clinical Opiate Withdrawal Scale (COWS) monitoring. The protocol for the COWS is an 11-item scale designed to be administered by a clinician. This tool can be used in both inpatient and outpatient settings to reproducibly rate common signs and symptoms of opiate withdrawal and monitor these symptoms over time.

Gastroenterology was consulted due to nausea, vomiting, and abdominal pain. The patient was noted to be sitting in bed eating a regular diet: he stated that for the last 24 hours, he had been able to tolerate oral intake. He further reported that generalized abdominal pain was improving. The patient was placed on a proton pump inhibitor and hydroxyzine PRN. Given opioid use, he admitted to constipation and was placed on MiraLAX (Bayer Healthcare, Indianola, PA).

On day 2, one of two sets of blood cultures returned positive for gram-negative rods, and the patient was started on 1 g IV twice daily of cefepime. The lab ruled the initial positive blood culture an error, and the patient was stopped on antibiotics. A repeat blood culture was obtained. The urine culture resulted in a negative result. The patient remained afebrile and normotensive.

Nephrology was consulted, and it was noted that the sodium had increased from 123 to 129 mEq/L (reference range: 136-145 mEq/L) over the course of 12 hours. To limit the overcorrection of hyponatremia, the patient was given a dose of desmopressin acetate (DDAVP) in the morning and another dose in the evening. DDAVP is a synthetic analog (1‐deamino‐8‐D‐arginine vasopressin) of the antidiuretic hormone L‐arginine‐vasopressin with hemostatic properties. It was noted that given urine osmolality of 243 mmol/kg (reference: 50-1,200 mmol/kg), the patient was appropriately mobilizing free water. Hypokalemia improved with potassium increasing from 2.2 to 3.0 mEq/L (reference: 3.5-5.0 mEq/L). With the replacement of potassium, the patient’s metabolic alkalosis also improved, and the bicarbonate level was noted to be steadily decreasing, down to 32 mEq/L (reference: 23-26 mEq/L). Urine pH at 8.5 also denoted appropriate renal elimination of bicarbonate. The patient's phosphorus level was low at 1.2 mg/dL (reference: 2.4-4.1 mg/dL), consistent with poor nutritional intake before admission. Overall, good oral fluid intake and serial monitoring of sodium were recommended.

By day 4, the patient generally reported continued symptomatic improvement with no recurrence of nausea, vomiting, or abdominal pain. Sodium and potassium had been corrected completely, along with the patient’s metabolic alkalosis. The patient was noted to be in stable condition from both a nephrology and infectious disease standpoint.

Despite the general lack of symptoms, it was decided to obtain a hepatitis C virus (HCV) antibody lab due to his history of IV drug use and the abdominal pain he had on admission. He was found to be HCV antibody positive. On discharge, a referral was given to outpatient gastroenterology for further workup. He was referred to a Suboxone Clinic and drug rehabilitation facility for follow-up. The repeat blood culture was noted to be negative. The patient was discharged in stable condition.

## Discussion

This case illustrates the importance of keeping a wide differential in mind, particularly those with a history of substance use. Of note, the patient had physical examination findings consistent with endocarditis. However, an echocardiogram ruled out this diagnosis. The patient also was incidentally noted to have hepatitis C despite being asymptomatic throughout the presentation. The patient’s history of heroin use was critical while determining which tests to order. Comprehensive barrier precautions, including the use of plastic aprons, face protection, and water-resistant gowns, as well as double-gloving, can significantly reduce blood exposure by 87% and the volume of transmitted blood by 95%, thus lowering the potential viral load from an infected patient [3].

Furthermore, this case depicts the need for prompt workup. Timely diagnosis and treatment are critical to prevent poor outcomes, including death. The case highlights the critical need for prompt and thorough diagnostic workup. Quick identification and treatment are essential to prevent adverse outcomes, including death. In this instance, the patient's substance use history was pivotal in guiding the diagnostic process.

Substance abuse can lead to vomiting, which may result in dangerous electrolyte imbalances. Drugs can disrupt normal oral intake, excretion, and overall body electrolyte distribution. For this patient, hyponatremia [4] and hypokalemia with metabolic alkalosis [5] were notable findings. Correcting these imbalances requires careful monitoring to avoid complications like overcorrection [6]. In this case, overcorrection of hyponatremia was managed with DDAVP, underscoring the importance of close and frequent electrolyte monitoring, ideally every 12 hours [7].

While immediate concerns such as electrolyte imbalances must be managed, long-term strategies include detoxification and rehabilitation to prevent the recurrence of such medical issues. Ensuring that the patient receives comprehensive care, including substance use treatment, is crucial for their overall recovery and health maintenance.

## Conclusions

This case of hepatitis C presenting as endocarditis in a young patient with substance use disorder underscores the intricate link between intravenous drug use and severe infectious complications. The rising incidence of endocarditis among younger populations with substance use disorders highlights the necessity for heightened clinical awareness and comprehensive prevention strategies. Healthcare providers should prioritize harm reduction measures, such as promoting safe injection practices and providing access to addiction treatment services. These efforts are vital to reducing the morbidity and mortality associated with endocarditis and other drug-related infections​.
